# Intestinal Alkaline Phosphatase Prevents Sulfate Reducing Bacteria-Induced Increased Tight Junction Permeability by Inhibiting Snail Pathway

**DOI:** 10.3389/fcimb.2022.882498

**Published:** 2022-05-26

**Authors:** Sudha B. Singh, Cristina N. Coffman, Matthew G. Varga, Amanda Carroll-Portillo, Cody A. Braun, Henry C. Lin

**Affiliations:** ^1^ Biomedical Research Institute of New Mexico, New Mexico Veterans Affairs (VA) Health Care System, Albuquerque, NM, United States; ^2^ Division of Gastroenterology and Hepatology, Department of Medicine, University of New Mexico, Albuquerque, NM, United States; ^3^ Medicine Service, New Mexico Veterans Affairs (VA) Health Care System, Albuquerque, NM, United States

**Keywords:** *Desulfovibrio vulgaris*, snail, transepithelial electrical resistance (TEER), intestinal alkaline phosphatase (IAP), occludin

## Abstract

Tight junctions (TJs) are essential components of intestinal barrier integrity and protect the epithelium against passive paracellular flux and microbial translocation. Dysfunctional TJ leads to leaky gut, a condition associated with diseases including inflammatory bowel disease (IBD). Sulfate-Reducing Bacteria (SRB) are minor residents of the gut. An increased number of *Desulfovibrio*, the most predominant SRB, is observed in IBD and other diseases associated with leaky gut. However, it is not known whether *Desulfovibrio* contributes to leaky gut. We tested the hypothesis that *Desulfovibrio vulgaris* (DSV) may induce intestinal permeability *in vitro*. Snail, a transcription factor, disrupts barrier function by affecting TJ proteins such as occludin. Intestinal alkaline phosphatase (IAP), a host defense protein, protects epithelial barrier integrity. We tested whether DSV induced permeability in polarized Caco-2 cells *via* snail and if this effect was inhibited by IAP. Barrier integrity was assessed by measuring transepithelial electric resistance (TEER) and by 4kDa FITC-Dextran flux to determine paracellular permeability. We found that DSV reduced TEER, increased FITC-flux, upregulated snail protein expression, caused nuclear translocation of snail, and disrupted occludin staining at the junctions. DSV-induced permeability effects were inhibited in cells knocked down for snail. Pre-treatment of cells with IAP inhibited DSV-induced FITC flux and snail expression and DSV-mediated disruption of occludin staining. These data show that DSV, a resident commensal bacterium, can contribute to leaky gut and that snail may serve as a novel therapeutic target to mitigate DSV-induced effects. Taken together, our study suggests a novel underlying mechanism of association of *Desulfovibrio* bloom with diseases with increased intestinal permeability. Our study also underscores IAP as a novel therapeutic intervention for correcting SRB-induced leaky gut *via* inhibition of snail.

## Introduction

Intestinal epithelium is a formidable barrier that protects the host against luminal bacteria, harmful molecules, as well as pathogens. Barrier integrity is mainly dependent on tight junctions (TJs) that include proteins such as claudins, occludin, zonula occludens, adheren junctions (AJs) composed of cadherins and catenins, desmosomes comprised of desmogleins and desmocollins, and gap junctions. Together, these junctions play an important role in preventing paracellular flux and microbial translocation (Wells et al., 2017). Dysfunction of barrier integrity with increased intestinal permeability (leaky gut) has been linked to many diseases associated with gut microbial dysbiosis such as IBD, Parkinson’s disease, and metabolic syndrome (Degruttola et al., 2016; Yu, 2018; Metta et al., 2021). Sulfate reducing bacteria (SRB) are residents of the gut found mainly in the colon in humans and in animals (Nava et al., 2012). SRB are usually minor members of the gut but are often found in increased numbers (bloom) in several conditions associated with leaky gut such as IBD. Among SRB, *Desulfovibrio* is the most prominent genus (Loubinoux et al., 2002). *Desulfovibrio* spp. have been reported to increase in number in various intestinal and extraintestinal diseases such as IBD, pouchitis, periodontitis, and Parkinson’s disease (Singh and Lin, 2015; Murros et al., 2021). While the pathological role of *Desulfovibrio* spp. has been studied mainly in the context of their effect on inflammatory pathways (Weglarz et al., 2003; Kushkevych et al., 2019; Singh et al., 2021), whether these bacteria induce leaky gut is not known even though leaky gut is associated with several diseases related to DSV bloom (Loubinoux et al., 2002). Snail is a transcription factor responsible for increased intestinal permeability (Forsyth et al., 2011; Elamin et al., 2014; Liu et al., 2020) by negatively regulating TJ proteins such as occludin, claudins, ZO-1 as well as AJ proteins such as E-cadherin (Cano et al., 2000; Martinez-Estrada et al., 2006). SiRNA against snail was shown to inhibit tight junction permeability induced by various experimental stimuli (Elamin et al., 2014; Zhou et al., 2018; Liu et al., 2020). Snail expression has been reported to be upregulated by pathogens such as *Salmonella*, Group B *Streptococcus* (Kim et al., 2015) and *H. pylori* (Marques et al., 2018; Liu et al., 2020) but the effect of resident commensal bacteria such as *Desulfovibrio* on snail is not known. Intestinal alkaline phosphatase (IAP) is a host defense protein produced by enterocytes in the small intestine and is secreted into the intestinal lumen, blood, and stool. Several protective functions have been attributed to IAP including lipopolysaccharide (LPS) detoxification, regulation of bicarbonate secretion in the small intestine, regulation of gut microbiome, dephosphorylation of proinflammatory nucleotides (Fawley and Gourlay, 2016; Bilski et al., 2017; Lalles, 2019), and induction of anti-inflammatory autophagy (Singh et al., 2020). IAP has also been reported to regulate tight junction proteins and to protect against LPS-induced barrier dysfunction (Liu et al., 2016). In this study, we tested the hypothesis that DSV may induce intestinal permeability in Caco-2 cells in a snail-dependent manner and this effect may be reversed by IAP.

## Materials and Methods

### Cell Culture and Treatments

Human colonic epithelial cells Caco-2 were purchased from ATCC (Manassas, VA). Cells were grown in DMEM+20% FBS+ Penicillin and Streptomycin (Thermo Fisher Scientific, Waltham, MA). Cells were grown at 37°C in a humidified incubator with 5% CO_2._ To allow polarization and differentiation, cells were seeded at a density of 5x10^5^ cells/well in 12-well 0.4 μm trans-well inserts for 3 weeks. Medium was replaced every 2-3 days. Twenty-four hours before infection, growth medium was replaced with colorless medium (DMEM+20% FBS) without antibiotics. Cells were treated with DSV at various multiplicity of infection (MOI) for 24 hours. DSV was added to the apical surface. For IAP treatment, cells were incubated with 500U/ml of IAP (Sigma-Aldrich, St. Louis, MO) or vehicle alone (solution in which IAP was supplied) at the apical and at basolateral surface for 24 hours prior to DSV challenge.

### *Desulfovibrio vulgaris* (DSV) Growth

*Desulfovibrio vulgaris* Hildenborough (ATCC 29579, Manassas, VA) was grown anaerobically in Hungate tubes using Postgate’s organic liquid medium. Media composition: 10.56 mM Na_2_SO_4_, 13.29 mM MgSO_4_, 4.12 mM L-Cysteine, 0.4% sodium lactate (60% syrup), 0.4% yeast extract, and 0.5% tryptone. Cultures were grown for ~24 hours in 5 ml aliquots at 37°C. Bacteria were counted using Quantom Tx cell counter (Logos Biosystems, South Korea) and a Petroff Hausser counting chamber (Hausser Scientific). Before infection, bacteria were pelleted (6000 rpm for 5 mins) and resuspended in sterile phosphate buffered saline solution (PBS). For heat-killed bacteria (HK), DSV was autoclaved and the volume equivalent to the original count was used for infections. For obtaining bacterial culture supernatant, live DSV were centrifuged and the supernatant was filtered through a 0.2 μm filter to remove any remaining bacteria. 100 μl of this supernatant was added to Caco-2. As a control for this, 100 μl of Postgate’s medium was added to the cells. Cells were infected with live, HK, or supernatant for 24 hours.

### TEER and FITC Flux

Tight junction barrier integrity in 21- day old polarized Caco-2 cells grown in 12-well trans-well plates was assessed *via* transepithelial electric resistance (TEER) using EVOM meter (WPI, Sarasota, FL). Cells with TEER >450 Ω were used in the experiments. Paracellular permeability was quantified by measuring FITC-Dextran flux. Fluorescein isothiocyanate–dextran 4000 (FITC-Dextran) FD-4 was procured from Sigma-Aldrich. A solution of 25 mg/ml of FD-4 was made in sterile PBS. 40μl of this solution was added apically to the cells post infection. After 1 hr. of incubation, 100 μl of the basolateral medium was collected and analyzed for the presence of FITC fluorescence. Fluorescence was measured at the wavelengths of excitation and emission at 485 and 520 nm, respectively using a Synergy HTX Multi-Mode Reader (Biotek, Winooski, VT). All the tests were carried out in triplicates.

### siRNA Transfection

SiRNA#1 ID was s13185 Ref Seq NM_005985.39 (siRNA location 1211). SiRNA#2 ID was s13186 Ref Seq NM_005985.3(siRNA location 832). Cells were transfected with either control scrambled (Scr) or with Snail silencer select siRNAs (35nM) with Lipofectamine RNAiMax using the manufacturer’s instructions. Briefly, cells (1 × 10^5^/well) were seeded into a 12-well trans-well plate and grown for 21 days. siRNAs mixed with lipofectamine RNAiMAX in OptiMEM medium were added to the apical surface of the trans-well for 48 hours. First, transfection efficiency was measured by using two different siRNAs against snail, either alone or in combination. Transfection efficiency was measured by western blotting to assess the levels of snail protein. After 48 hours, cells were infected with DSV (MOI20) for 24 hours. Permeability was assessed by measuring FITC-Dextran flux. Cells were lysed and analyzed for snail expression by western blot.

### Western Blot

Cells were lysed in Lysis buffer (Thermo Fisher Scientific: #87787) containing protease and phosphatase inhibitors (Thermo Fisher Scientific: #1861281) for 30 mins at 4°C with shaking. Lysates were centrifuged at 12,000 rpm for 5 mins at 4°C and supernatants were collected. Protein concentration in the supernatants was determined with Bradford reagent (Bio-Rad Laboratories, Hercules, CA). 50 µg of protein samples were run on SDS-PAGE (4-20% tris-glycine) and transferred to nitrocellulose membranes. Membranes were blocked in 5% milk in PBS-T (0.1%Tween 20) for 30 mins followed by overnight incubation in antibodies against Actin (Cell Signaling Technology: 4970) and snail (Cell Signaling Technology: #3879) Antibodies were diluted as recommended by the manufacturer. Blots were incubated with secondary antibodies (Cell Signaling Technology: #7074) at room temperature for 1 hour (dilution of 1:2000) and developed using enhanced Chemiluminescence HRP signal (Thermo Fisher Scientific: #34577).

### Immunofluorescence

Polarized Caco-2 cells were fixed with 4% paraformaldehyde for 15 mins. Trans-wells were then washed apically and basolaterally with PBS 3 times for 5 mins each. Cells were blocked in a blocking solution consisting of 5% FBS and 0.3% Triton-X 100 for 1 hour. This was followed by incubation of both apical and basolateral surface of the trans-wells with primary antibody against snail1 or occludin (Cell Signaling Technology: 91131) at 4°C. Cells were then washed with PBS followed by incubation with secondary anti-rabbit antibody (Thermo Fisher Scientific: #A21206) for 2 hours at room temperature. Imaging was done with Olympus confocal microscope using Z-stacks. Final images were presented as compressed Z-stack images to represent the entire depth of the monolayer for an unbiased view of the tight junctions.

### Statistical Analysis

All graphs were generated using GraphPad Prism 8 (GraphPad Software, San Diego, CA). Data represents Mean ± SEM from at least three independent experiments. Values were normalized to control and presented as the percent of change relative to Control set at 100%. Three or more groups were compared with one-way ANOVA. Two groups were compared with students t-test for statistical analysis. P values <0.05 were considered statistically significant.

## Results

### DSV Decreased TEER and Increased Paracellular Permeability in Polarized Caco-2 Cells

**Figure 1:**
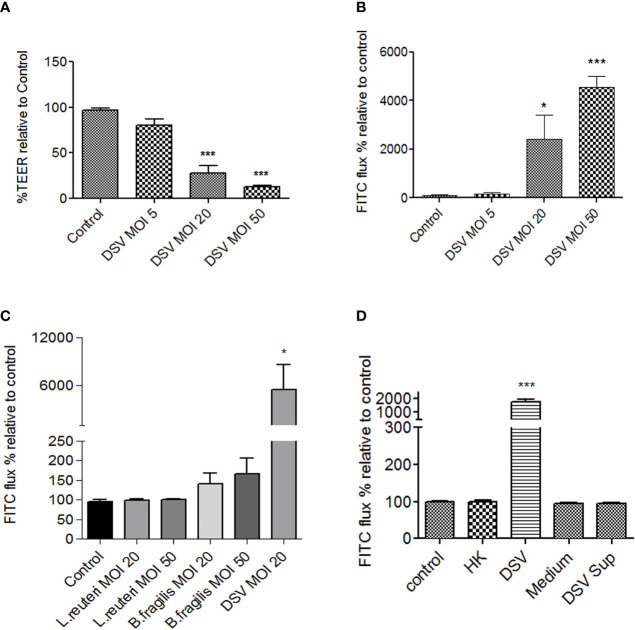
DSV decreased TEER and increased permeability in polarized Caco-2 cells. Polarized Caco-2 cells were treated with DSV with various MOIs for 24 hours. **(A)** TEER was measured using EVOM ohmmeter by inserting electrodes into the trans-wells. **(B)** Cells were incubated for 1hr with 40μl 4kDa FITC-Dextran (25mg/ml) added to the apical surface. After 1 hr. 100μl of the medium from basolateral side was removed and analyzed for FITC fluorescence, using excitation and emission at 485nm and 520nm, respectively. **(C)** FITC-Dextran flux was monitored in cells infected with *L.reuteri*, *B.fragilis*, or DSV for 24 with different MOIs. **(D)** FITC-dextran flux in cells were infected with live, heat killed (HK), bacterial Postgate’s culture medium alone, or with filtered culture supernatant of live DSV bacteria. Graph in all panels represent Mean ± SEM from at least 3 independent experiments. *p<0.05, ***p<0.001.

We first tested whether DSV induced increased intestinal permeability in Caco-2 cells by measuring transepithelial electrical resistance (TEER) and FITC-Dextran flux. Polarized Caco-2 cells were treated with DSV using different multiplicity of infection (MOI) for 24 hours (**Figure 1**). We found that DSV challenge caused a significant reduction in TEER as % TEER at MOI 20 (27.88±8.26) and at MOI 50 (13.17±0.87) when compared to control uninfected cells (96.99±1.84, p<0.001) (**Figure 1A**). Next, we measured paracellular permeability by monitoring the flux of 4 kDa FITC-Dextran through the trans-well as a percentage relative to control. We found that MOI 20 (2418±979.9, p<0.05) and MOI 50 (4553±447.5, p<0.001) caused a significant increase in FITC-flux when compared to control cells (100.0±25.19)(**Figure 1B**). As MOI 20 was the lowest dose of DSV that caused significant increase in permeability, we used this dose for all subsequent experiments.

We further tested the effect of other bacteria such as those representing the most predominant phyla in the gut with gram negative Bacteroidetes (*B. fragilis*) and gram positive Firmicutes (*L. reuteri*) in comparison to DSV, on Caco-2 permeability by measuring FITC-Dextran flux. We found that DSV (5477±3083, p<0.05) but not *L. reuteri* or *B. fragilis*, even at a higher MOI 50, caused a significant increase in permeability compared to control (95.58±4.02)(**Figure 1C**). Thus, induction of intestinal permeability is not a universal feature of commensals and may be caused by selected opportunistic bacteria or pathobionts such as DSV that are found to bloom in various diseases.

Next, we tested whether increase in permeability was the property of live DSV or if it could also be induced by dead bacteria or by a secretory factor of these bacteria in the culture supernatant. Heat-killed DSV (HK) were added to Caco-2 cells (comparable to MOI 20 of live bacteria) for 24 hours. To obtain bacterial culture supernatant, live bacteria were pelleted and the supernatant was passed through a 0.2 μm filter to remove any bacteria in the supernatant. Cells were incubated with either the filtered supernatant (DSV Sup) or DSV growth medium alone as a control (Medium) for 24 hours. We found that only live DSV (1772±197.5, p<0.05) but not HK (100.1±3.32, p>0.05) or DSV Sup (94.91±1.88) increased the FITC-flux when compared to control (100±1.71) (**Figure 1D**) suggesting that induction of permeability could only be caused by live bacteria and was not a structural property of the bacteria or due to bacterial secretory product(s).

### DSV Induced Changes in the Localization of TJ Protein Occludin

**Figure 2:**
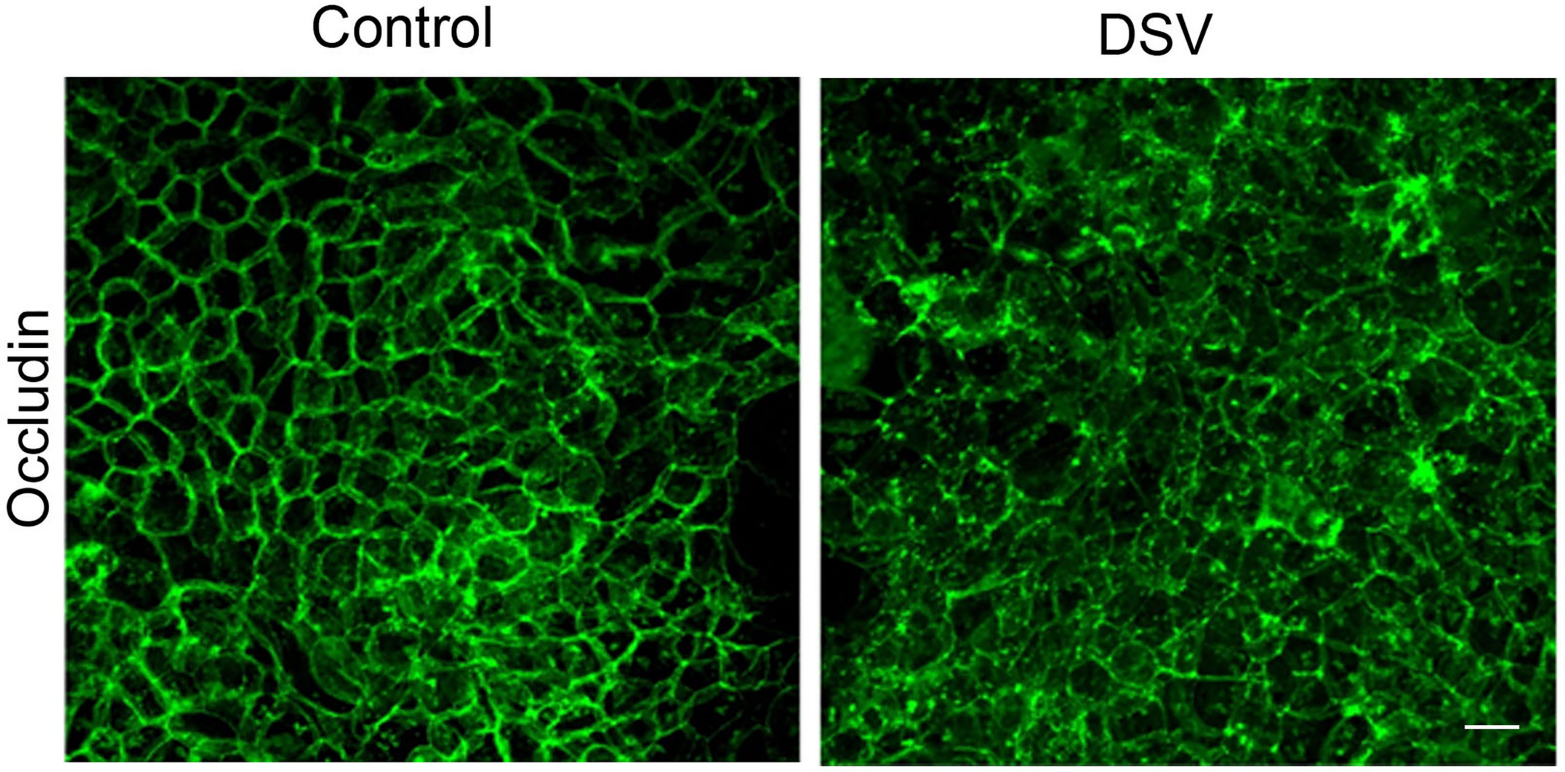
DSV induced changes in the localization of Occludin. Polarized Caco-2 cells were infected with DSV (MOI 20) for 24 hours. Cells were then fixed with 4% paraformaldehyde. After blocking in solution containing 5% FBS, 0.3% TritonX-100 in PBS, cells were incubated with anti-occludin antibody (in 1% BSA with 0.1% TritonX-100 in PBS) overnight at 4˚C. Cells were washed with PBS and incubated further with secondary anti-rabbit Alexa Flour-488 antibody for 2 hours at room temperature. Cells were washed and mounted using Prolong Gold (Thermo Fisher). Cells were visualized with Olympus Fluoview FV1200 confocal microscope. Z-stacks were performed for each image and the final images were collected after compressing the z-stacks together.

We next investigated the mechanism of DSV-induced permeability. We first analyzed the protein expression of tight junction proteins such as occludin and claudin-2. However, western blot analysis did not show a downregulation of these proteins in response to DSV (data not shown). We therefore analyzed changes in localization by immunofluorescence and found that while DSV did not inhibit protein expression of occludin, it caused a remarkable shift in the localization of this protein (**Figure 2**) from a distinct and sharp staining of paracellular spaces to a loss of this localization with disorganized displacement to the cytoplasm. Thus, loss of discrete localization of occludin from the paracellular space explains the increased permeability that represents impaired barrier function. Increase in TJ permeability by DSV was unlikely due to cell death, as we did not detect a decrease in total protein concentration in cells treated with DSV at 24 hours when compared to control cells, as measured by Bradford protein estimation assay. In addition, we found that AJ protein E-cadherin did not show any changes in protein expression or localization in response to DSV(data not shown). These findings argue away from the possibility of cell death by DSV.

### DSV Induced Snail Protein Expression

**Figure 3:**
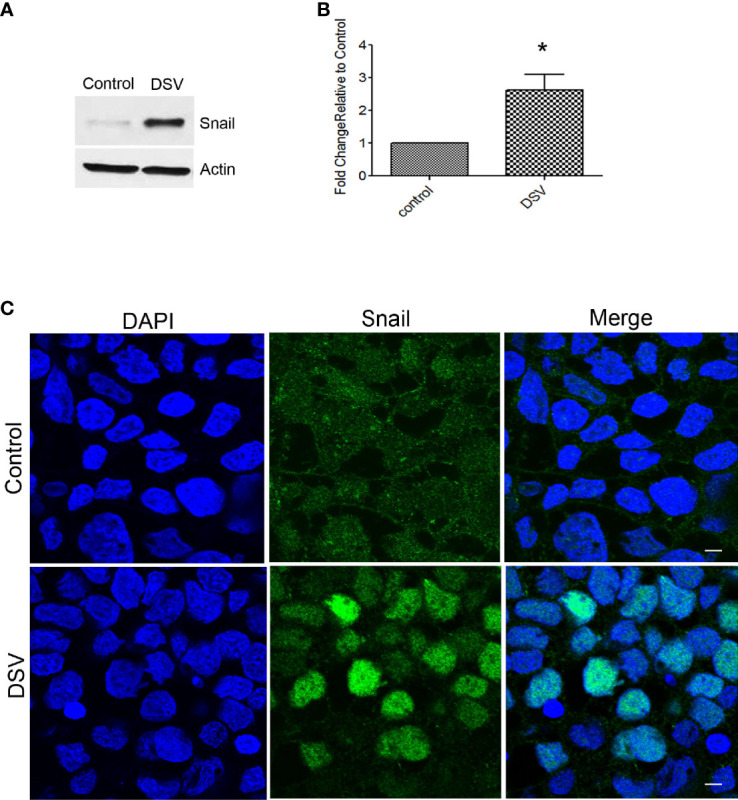
DSV induced snail protein expression. Polarized cells were treated with DSV (MOI 20) for 24 hours. **(A, B)** snail detection by Western blot in Control and DSV-treated cells. Actin was used a loading control. Graph represents Mean ± SEM from at least 3 independent experiments. *p<0.05.**(C)** Immunofluorescence of cells infected with or without DSV. Cells were fixed with 4% paraformaldehyde and stained with anti-snail antibody followed by secondary antibody labeled with Alexa Flour–488. Cells were visualized with Olympus Fluoview FV1200 confocal microscope. Scale bar=20 µm.

Snail is a transcription factor that disrupts barrier integrity by downregulating the expression of tight junction proteins such as occludin in addition to its role in affecting adheren junction proteins such as E-cadherin. We tested whether DSV induced snail protein expression in Caco-2 cells. Compared to control cells, DSV-treated cells had a significant upregulation of snail protein expression (DSV: 2.62 ± 0.48 vs control: 1:00; p<.0.05) (**Figures 3A, B**). We also analyzed the nuclear translocation of snail in cells infected with DSV. We found that all control cells displayed a diffuse cytoplasmic snail staining and no nuclear staining (**Figure 3C**). However, after DSV infection, in ~50% cells, a strong nuclear staining of snail could be observed. In other DSV infected cells, we observed a faint signal in the nuclear snail. Thus, DSV induced snail protein expression and promoted its nuclear translocation.

### DSV Induced Barrier Permeability in a Snail-Dependent Fashion

**Figure 4:**
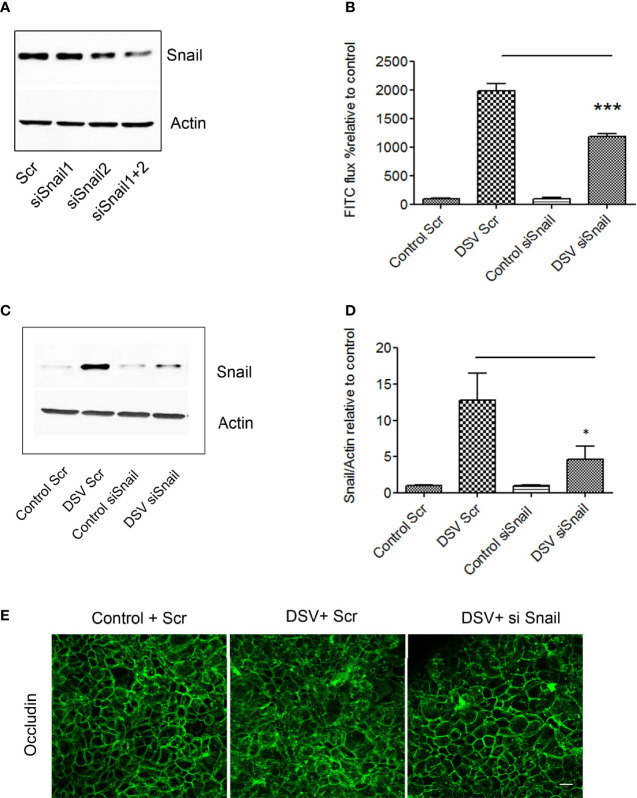
DSV induced permeability in snail-dependent fashion. Polarized Caco-2 cells were transfected with either scrambled control (Scr) siRNA or siRNA against snail (siSnail) for 48 hours. **(A)** transfection efficiency was determined by Western blotting in cells transfected with two siRNAs against snail alone or in combination. **(B)** Transfected cells were infected with DSV for further 24 hours. After 24 hours, FITC-Dextran was added on the apical side of the cells for 1 hr following which 100 μl of medium from the basolateral surface was collected and analyzed for FITC-flux. ***p < 0.001. **(C)** Snail expression was monitored by Western blotting in transfected cells challenged with DSV. **(D)** Graph represents Mean ± SEM from at least 3 independent experiments. *p<0.05. **(E)** Polarized Caco-2 cells were transfected with either Scr or Snail siRNA for 48 hours. This was followed by DSV infection for 24 hours. Cells were then fixed with 4% paraformaldehyde and probed by immunofluorescence for occludin staining as described in Materials and Methods. Images were collected as Z-stacks that were compressed at the end to generate the final images. Scale bar=20 µm.

As DSV caused an increase in snail protein expression and its nuclear translocation, we tested whether DSV-induced permeability was dependent on snail. For this, cells were first transfected with either scrambled siRNA (Scr) as control or with siRNA against Snail for 48 hours. To get the optimal transfection efficiency, we tested 2 siRNAs against snail independently or in combination (**Figure 4A**). We found that the best efficiency was obtained in the combined siRNA group. Based on these results, we used combination of 2 siRNAs to knock down snail in subsequent experiments (referred to as siSnail). Forty-eight hours after transfection, cells were infected with DSV for 24 hours. DSV induced marked increase in FITC-Dextran flux in cells transfected with Scr siRNA (DSV Scr: 1980±122.4 vs Control Scr: 100±4.02) (**Figure 4B**). However, in cells transfected with snail siRNA, we observed a significant decrease in FITC-Dextran flux after DSV infection when compared to DSV Scr infected cells (DSV+Scr:1980±122.4 vs DSV+siSnail: 1179± 60.42, p<0.001). Similarly, we also observed a significant reduction in snail expression in response to DSV in siSnail cells (4.52±1.96) when compared to DSV-infected Scr cells (12.78±3.65, p<0.05)(**Figures 4C, D**). Finally, we asked whether inhibition of snail could prevent disruption of occludin localization in response to DSV. Compared to Control+Scr cells, DSV+Scr cells had an obvious change in the staining of occludin from uniform cellular junction to a disorganized cytoplasmic staining (**Figure 4E**). However, in cells transfected with Snail siRNA, there was an inhibition in the disruption of paracellular staining of occludin in response to DSV when compared to DSV+Scr cells. Taken together, these results suggest that DSV-induced barrier permeability occurs in a snail-dependent manner.

### IAP Reversed DSV-Induced Permeability by Inhibiting Snail

**Figure 5:**
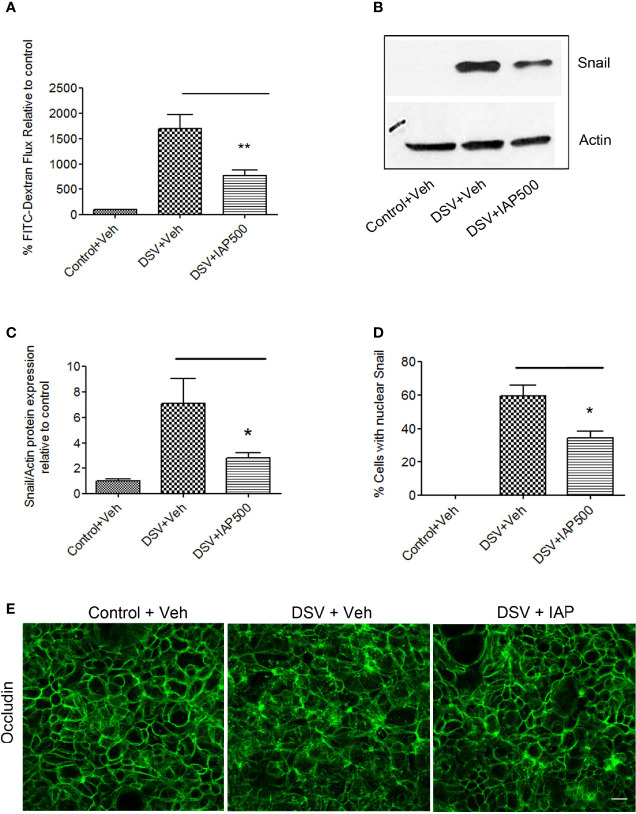
Intestinal Alkaline Phosphatase inhibits DSV-induced permeability by inhibiting snail. Polarized Caco-2 cells were pre-treated with IAP (500 U/ml) at the apical and the basolateral surface for 24 hours prior to challenge with DSV. **(A)** FITC-Dextran was added to the cells apically after 24 hours infection with DSV. Fluorescence was measured by taking out 100 μl medium from the basolateral surface after 1 hr. incubation. Graph represents Mean ± SEM of % FITC-flux compared to uninfected control cells from at least 3 independent experiments. **p < 0.01. **(B)** Snail protein expression was analyzed by Western blotting in cells pre-treated with or without IAP followed by infection with DSV. The solution in which IAP was supplied was used as a Vehicle control. Actin was used as a loading control. **(C)** Quantification of Western blot for detection of snail. Graph represents Mean ± SEM of ratio of Snail/Actin and values were normalized to control from at least 3 independent experiments. *p < 0.05. **(D)** Cells were pre-treated with or without IAP followed by infection with DSV. Cells were fixed with 4% paraformaldehyde and probed by immunofluorescence for snail staining as described in Materials and Methods. Graph represents quantification of % cells showing nuclear staining of snail in control, DSV or DSV+IAP treated groups. Values represent Mean ± SEM from three independent experiments. *p < 0.05. **(E)** Cells were pre-treated with or without IAP followed by infection with DSV. Cells were fixed with 4% paraformaldehyde and probed by immunofluorescence for occludin staining as described above. Images were collected as Z-stacks that were compressed at the end to generate the final images. Scale bar=20µm.

IAP is an important host defense protein secreted by the small intestinal cells and has a protective role in many diseases (Fawley and Gourlay, 2016). In addition to its other known protective functions, IAP has also been shown to protect the intestinal barrier *via* its effect on tight junction proteins such as occludin (Liu et al., 2016). We therefore tested whether IAP protected against DSV-induced permeability and whether it was through inhibition of snail. Cells were pre-treated with IAP (500U/ml) or with vehicle alone for 24 hours before infection with DSV (**Figure 5A**). We found that treatment of cells with IAP inhibited DSV-induced FITC-Dextran flux when compared to DSV+Veh treatment (DSV+IAP500: 770±115.8 vs DSV+Veh: 1697±276.7, p<0.01). Next, we analyzed induction of snail protein expression by DSV in the presence of IAP. Compared to DSV+Veh-treated cells (7.06 ± 1.98), there was a significant reduction in snail expression in cells pre-treated with IAP (DSV+IAP 500: 2.82 ± 0.42; p<0.05)(**Figures 5B, C**). We also analyzed nuclear localization of snail in response to DSV in the presence or absence of IAP (**Figure 5D**). Compared to DSV+Veh (59.45 ± 6.42), there was a significant reduction in the percentage of cells with nuclear snail in DSV+IAP treated cells (34.26 ± 4.06, p<0.05). Control cells had no nuclear snail staining. These results suggest that IAP inhibited DSV-induced snail expression and its nuclear localization. Finally, we analyzed the immunolocalization of occludin in DSV-treated cells in the absence or presence of IAP. We found that in DSV+Veh cells, there was a disruption in the cellular junction staining of occludin to a more cytoplasmic and diffuse staining pattern when compared to Control+Veh cells. However, in the presence of IAP, occludin staining appeared comparable to control, more at the junctions in DSV+IAP cells when compared to DSV+Veh cells (**Figure 5E**). Together, these data suggest that IAP inhibited DSV-induced permeability by inhibiting snail.

## Discussion

In this study, we report that *Desulfovibrio vulgaris* (DSV) induced increased barrier permeability in polarized Caco-2 cells. DSV belongs to the most abundant genus *Desulfovibrio* among SRB that is reported to overgrow in conditions associated with gut microbial dysbiosis such as IBD (Loubinoux et al., 2002). Recently, a few studies have shown that SRB may be the driving factors of adverse outcomes such as increased inflammation (Weglarz et al., 2003; Jia et al., 2017; Liu et al., 2020). Our group previously showed that administering DSV into the gut of mice caused impaired memory (Ritz et al., 2016) and slowed intestinal transit (Ritz et al., 2017). Recently, we showed that DSV induced Notch signaling pathway which, in turn, upregulated precursors of proinflammatory cascade (Singh et al., 2021). A recent report showed that a proinflammatory SRB *Fusobacterium nucleatum* induced increased intestinal permeability (Liu et al., 2020) suggesting that SRB may contribute to disease development by disrupting barrier function. Thus, there is a need to better understand the underlying mechanisms by which these bacteria act on the host. Such information may enable us to identify novel targets to control diseases associated with SRB bloom. In this study, we found that DSV increased barrier permeability *via* a novel mechanism of inducing the expression and nuclear translocation of the transcription factor snail. In addition, we showed that treatment with the host defense protein intestinal alkaline phosphatase (IAP) prevented DSV-induced barrier dysfunction by inhibiting snail induction by DSV. Depending upon their classification, resident commensal bacteria may affect barrier integrity differently. Studies have reported protective effects of probiotics such as *Lactobacillus* spp. by enhancing tight junction barrier (Han et al., 2019; Li et al., 2019). Similarly, bacteria such as *F. prausnitzii, R. intestinalis*, and *B. faecalis* have been shown to improve barrier function (Mohebali et al., 2020). In contrast, pathogens such as *Salmonella* and opportunistic commensals such as *E. coli* K12 (Bhat et al., 2019) induce intestinal permeability. Among SRB, *F. nucleatum*, a proinflammatory bacterium associated with various diseases such as periodontitis (Chaushu et al., 2012), cancer (Yang et al., 2017), and colitis (Liu et al., 2019), was found to induce barrier permeability *in vivo* and *in vitro* (Liu et al., 2020). *F. nucleatum* also inhibited the expression of ZO-1 and occludin in the colon and affected their distribution. In Caco-2 cells, *F. nucleatum* induced permeability as assessed by increased FITC-Dextran flux as well as by a loss of TEER. Our data of DSV-induced permeability extend these findings. Thus, SRB can cause harmful effects on the host intestinal barrier function in the setting of dysbiosis where SRB bloom is observed. We further dissected out the underlying mechanism of the adverse effect of DSV on barrier integrity and showed that DSV increased permeability by causing upregulation and nuclear translocation of the nuclear transcription factor snail. We also found that inhibition of snail by siRNA blocked DSV-induced permeability. Various studies have shown an increase in snail expression under experimental conditions in response to various harmful stimuli. Snail was found to be required for acetaldehyde-induced barrier disruption as well as redistribution of tight junction proteins ZO-1 and occludin (Elamin et al., 2014). LPS upregulated snail in several studies (Hong et al., 2015; Abdulkareem et al., 2018; Tavakolian et al., 2019). Snail expression is also upregulated by cytokines such as TGF- β (Valdes et al., 2002). In addition, inhibition of snail reversed alcohol-induced permeability in Caco-2 cells (Forsyth et al., 2011). Others studies have shown that infection with pathogenicbacteria increased snail expression and caused disruption of TJ proteins (Kim et al., 2015; Yang et al., 2016). In a study, Group B streptococcus (GBS) induced snail expression and disrupted occludin and ZO-1 in brain endothelium. There was also redistribution of TJPs at intercellular junctions, an effect not observed with heat-killed GBS. Expression of snail has been shown to be upregulated by *Salmonella typhimurium* where snail mediated downregulation of apical junction proteins E-cadherin and ZO-1 during infection (Liu et al., 2020). Overexpression of snail induced disruption of TJs and induced permeability in MDCK cells. Cells overexpressing snail also showed a decreased protein expression of E-cadherin and claudin-1 and redistribution of ZO-1 without affecting its protein expression (Martinez-Estrada et al., 2006). Redistribution of ZO-1 in the absence of change in protein expression is similar to our findings that DSV caused redistribution of occludin but did not affect its protein expression by Western blot. How DSV induces snail expression remains to be investigated. Pathways such as MAPK and TLR2/4 have been implicated in snail induction (Clarke et al., 2011), (Kim et al., 2015). Identification of bacterial factor(s) responsible for inducing permeability remains the subject of future studies.

We found that DSV caused disruption in the staining of occludin from the cellular junction to a more cytoplasmic and diffuse staining pattern without affecting its total protein expression as measured by western blot. A possible mechanism by which DSV may cause disruption of occludin localization at the junction is by potentially causing internalization of occludin by endocytosis mediated by clathrin-coated pits, caveolae, or *via* macropinocytosis. It was shown that occludin was endocytosed *via* macropinocytosis-like pathway as the underlying mechanism of barrier disruption in mouse model of alcoholic steatohepatitis *in vivo* and *in vitro* (Wang et al., 2019). In another study, IFN γ caused endocytosis of occludin by macropinocytosis in epithelial cells (Utech et al., 2005). Moreover, *Escherichia coli* cytotoxic necrotizing factor-1 (CNF-1) cause caveolae-mediated internalization of occludin in T84 epithelial cells, without affecting its protein expression (Hopkins et al., 2003). Using inhibitors of these three forms of endocytosis would shed light on whether DSV causes internalization of occludin *via* endocytosis. While the redistribution of occludin by DSV may occur independent of changes in its protein expression, it is possible that changes in protein expression of occludin can occur at a time later than 24 hours, downstream of changes in its redistribution by DSV. Further studies will be aimed at analyzing these possibilities.

As a potential treatment of DSV-induced barrier permeability, we tested whether IAP inhibited DSV-induced permeability. IAP is a host defense protein with many protective functions (Estaki et al., 2014; Lalles, 2014; Bilski et al., 2017) including its role in maintaining barrier integrity. Mouse embryonic fibroblasts derived from IAP-KO mice had lower levels of tight junction proteins (TJPs) ZO-1, ZO-2, and occludin and Caco-2 and T84 cells overexpressing IAP had elevated levels of the TJPs (Liu et al., 2016). In a study, IAP supplementation was found to reduce intestinal permeability and suppress gut bacterial translocation in the blood stream, liver, and lung in a mouse model of peritonitis, and also enhance transepithelial resistance and decrease claudin-2 expression in Caco-2 cells (Wang et al., 2015). Treatment with IAP also reversed the increased permeability in the small intestine in a CLP mouse model (Plaeke et al., 2020). Oral supplementation with IAP prevented alcohol-induced gut barrier dysfunction and TJ protein loss (Hamarneh et al., 2017). IAP-KO mice had increased gut permeability and decreased levels of TJ proteins ZO-1, ZO-2, occludin, and claudin1 when compared to WT mice (Hamarneh et al., 2014). IAP was also found to protect intestinal barrier function in aged mice (Kuhn et al., 2020). Thus, abundant evidence supports the role of IAP in protecting the barrier and thereby preventing the translocation of bacteria, among other harmful effects. In this study, we tested a previously used dose of IAP (500U/ml) in Caco-2 cells in a study where IAP prevented down-regulation and mis-localization of TJ proteins such as occludin in response to LPS and prevented LPS-induced barrier dysfunction (Liu et al., 2016). In accordance with these findings, we observed that IAP reversed DSV-induced barrier dysfunction. In addition, we demonstrated that IAP inhibited DSV-induced increase in snail protein expression and its nuclear translocation. IAP also corrected the mis-localization of occludin caused by DSV. How IAP inhibits DSV-induced snail remains to be investigated. In our previous study, we reported that IAP induced autophagy in epithelial cells (Singh et al., 2020). As autophagy protects the barrier function (Nighot et al., 2015; Nighot and Ma, 2016) and also targets snail for degradation (Grassi et al., 2015; Zou et al., 2017; Zada et al., 2019), it is possible that protective effects of IAP are mediated through autophagy. Future studies will be aimed at understanding the underlying mechanism of how IAP protects against DSV-induced snail expression as well as DSV-induced permeability.

Limitations of our study are that we do not have *in vivo* data to extend our *in vitro* findings and we did not examine other members of TJ proteins such as claudin1 that has been shown to be inhibited by snail (Martinez-Estrada et al., 2006). Subsequent studies will include expansion of our findings into *in vivo* models and examination of other TJ proteins inhibited by snail to better understand the mechanisms of DSV-induced tight junction permeability. In addition, while IAP inhibited DSV-induced snail expression and localization and also inhibited DSV-induced permeability (**Figure 5**), further studies are needed to unequivocally prove that protective effects of IAP on DSV-induced permeability are mediated *via* its inhibition of snail. Future studies will also focus on the kinetics of snail translocation to the nucleus as well as the redistribution and mechanism of occludin localization in response to DSV.

In conclusion, our study demonstrates that DSV induced a loss of epithelial barrier integrity and caused an aberrant localization of TJ protein occludin in a snail-dependent manner. IAP prevented DSV-induced permeability, prevented induction of snail expression and its nuclear translocation by DSV, and corrected the localization of occludin. Thus, our study identifies DSV as a contributor to the disease phenotype of leaky gut and identifies snail as a novel therapeutic target of DSV-induced barrier permeability. Our study also underscores IAP as a novel therapeutic intervention for correcting SRB-induced leaky gut *via* inhibition of snail. As many inflammatory diseases are linked to SRB overgrowth and leaky gut, it would be worthwhile to test whether IAP intervention would correct the phenotypes of leaky gut associated with SRB bloom.

## Data Availability Statement

The original contributions presented in the study are included in the article/supplementary material. Further inquiries can be directed to the corresponding author.

## Author Contributions

SS designed and performed experiments, analyzed the data, and wrote the manuscript; CC and MV performed the experiments; AC-P and CB helped with experimental design and setup and; HL conceptualized and designed the study and edited the manuscript. All authors contributed to the article and approved the submitted version.

## Funding

This study was funded by the Winkler Bacterial Overgrowth Research Fund: BRINM-217.

## Conflict of Interest

The authors declare that the research was conducted in the absence of any commercial or financial relationships that could be construed as a potential conflict of interest.

## Publisher’s Note

All claims expressed in this article are solely those of the authors and do not necessarily represent those of their affiliated organizations, or those of the publisher, the editors and the reviewers. Any product that may be evaluated in this article, or claim that may be made by its manufacturer, is not guaranteed or endorsed by the publisher.
